# STOP-Bang Score and Prediction of Severity of Obstructive Sleep Apnea in a First Nation Community in Saskatchewan, Canada

**DOI:** 10.3390/clockssleep4040042

**Published:** 2022-10-12

**Authors:** James A. Dosman, Chandima P. Karunanayake, Mark Fenton, Vivian R. Ramsden, Jeremy Seeseequasis, Delano Mike, Warren Seesequasis, Marie Neubuhr, Robert Skomro, Shelley Kirychuk, Donna C. Rennie, Kathleen McMullin, Brooke P. Russell, Niels Koehncke, Sylvia Abonyi, Malcolm King, Punam Pahwa

**Affiliations:** 1 Canadian Centre for Health and Safety in Agriculture, University of Saskatchewan, 104 Clinic Place, Saskatoon, SK S7N 2Z4, Canada; 2 Department of Medicine, University of Saskatchewan, 103 Hospital Drive, Saskatoon, SK S7N 0W8, Canada; 3 Division of Respirology, Critical Care, and Sleep Medicine, College of Medicine, University of Saskatchewan, 104 Clinic Place, Saskatoon, SK S7N 2Z4, Canada; 4 Department of Academic Family Medicine, University of Saskatchewan, West Winds Primary Health Centre, 3311 Fairlight Drive, Saskatoon, SK S7M 3Y5, Canada; 5 Willow Cree Health Services, Beardy’s & Okemasis Cree Nation, P.O. Box 96, Duck Lake, SK S0K 1J0, Canada; 6 Beardy’s & Okemasis Cree Nation Band, P.O. Box 340, Duck Lake, SK S0K 1J0, Canada; 7 College of Nursing, University of Saskatchewan, 104 Clinic Place, Saskatoon, SK S7N 2Z4, Canada; 8 Department of Community Health & Epidemiology, College of Medicine, University of Saskatchewan, 107 Wiggins Road, Saskatoon, SK S7N 5E5, Canada

**Keywords:** STOP-Bang score, obstructive sleep apnea, First Nations, obesity, snoring

## Abstract

The STOP-Bang questionnaire is an easy-to-administer scoring model to screen and identify patients at high risk of obstructive sleep apnea (OSA). However, its diagnostic utility has never been tested with First Nation peoples. The objective was to determine the predictive parameters and the utility of the STOP-Bang questionnaire as an OSA screening tool in a First Nation community in Saskatchewan. The baseline survey of the First Nations Sleep Health Project (FNSHP) was completed between 2018 and 2019. Of the available 233 sleep apnea tests, 215 participants completed the STOP-Bang score questionnaire. A proportional odds ordinal logistic regression analysis was conducted using the total score of the STOP-Bang as the independent variable with equal weight given to each response. Predicted probabilities for each score at cut-off points of the Apnea Hypopnea Index (AHI) were calculated and plotted. To assess the performance of the STOP-Bang questionnaire, sensitivity, specificity, positive predictive values (PPVs), negative predictive values (NPVs), and area under the curve (AUC) were calculated. These data suggest that a STOP-Bang score ≥ 5 will allow healthcare professionals to identify individuals with an increased probability of moderate-to-severe OSA, with high specificity (93.7%) and NPV (91.8%). For the STOP-Bang score cut-off ≥ 3, the sensitivity was 53.1% for all OSA and 72.0% for moderate-to-severe OSA. For the STOP-Bang score cut-off ≥ 3, the specificity was 68.4% for all OSA and 62.6% for moderate-to-severe OSA. The STOP-Bang score was modestly superior to the symptom of loud snoring, or loud snoring plus obesity in this population. Analysis by sex suggested that a STOP-Bang score ≥ 5 was able to identify individuals with increased probability of moderate-to-severe OSA, for males with acceptable diagnostic test accuracy for detecting participants with OSA, but there was no diagnostic test accuracy for females.

## 1. Introduction

Obstructive sleep apnea (OSA) is a common sleep-related breathing disorder. The prevalence of OSA is increasing worldwide [1,2,3]. The prevalence and severity of OSA are associated with obesity, age, sex, lifestyle and ethnicity [4,5,6]. Information about OSA among First Nations in Canada is sparse. The gold standard for the diagnosis of OSA is polysomnography (PSG) conducted overnight in a sleep laboratory. Because of cost and availability of polysomnography, a number of clinical screening tools have been developed for the purpose of identifying high risk patients for diagnosis and treatment.

The STOP-Bang questionnaire is an easy to administer scoring model consisting of eight questions [7]. The questionnaire includes four yes/no clinical options: (STOP: snoring, tiredness, observed apnea and high blood pressure) and four demographic options: (BANG; BMI, age, neck circumference and gender) questions. The STOP-Bang questionnaire was originally validated in a surgical population at preoperative clinics, but it has been extensively used in the other settings and in the general population worldwide [8]. A recent meta-analysis reported that the STOP-Bang is a valid and effective screening tool for OSA in the general population and commercial drivers [9]. In the surgical setting, a score ≥ 3 has shown a high sensitivity for identifying OSA (93% for moderate OSA and 100% for severe OSA) [10]. Therefore, a score ≥ 3 cut off is considered helpful to detect patients having moderate and severe OSA. However, the specificity at the same cut-off is low (47% for moderate OSA and 37% for severe OSA), which can result in high false-positive rates [7]. The STOP-Bang questionnaire has not been used with First Nations peoples previously. The objective of this paper was to determine the predictive parameters and the utility of the STOP-Bang questionnaire as an OSA screening tool in a First Nation community in Saskatchewan.

## 2. Results

**Figure 1:**
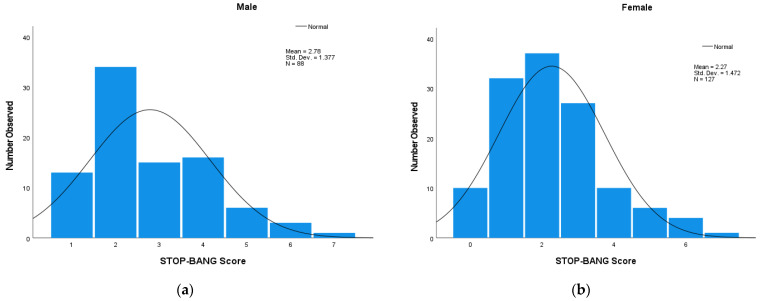

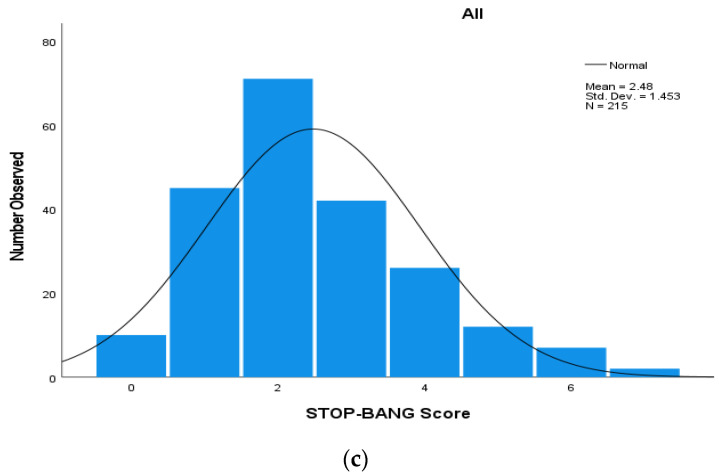
Distribution of the STOP-Bang summary scores. (**a**) Distribution of the STOP-Bang summary scores for males. (**b**) Distribution of the STOP-Bang summary scores for females. (**c**) Distribution of the STOP-Bang summary scores for all.

The participation in a Level 3 one-night home sleep test was 56% (233/418) of those who completed the survey questionnaire in Community A. There were 233 sleep apnea tests available and of those participants 215 STOP-Bang scores were available. The mean duration for the Apnea Link evaluation was 356 min (SD = ±135 min). There were 183 evaluations greater than or equal to 240 min and 50 evaluations were less than 240 min. In these two groups, mean AHI and demographic characteristics such as mean age and mean BMI were not significantly different; however, more males were found in the less than 240 min group and more females were found in the greater than or equal to 240 min group (*p* = 0.054). The distributions of the STOP-Bang summary scores for males, females and the entire group are shown in Figure 1a–c. In Figure 1, standard derivations are close to the mean; however, they are lower than the standard deviations in Figure 1a–c. This indicates that in all three figures measured, the STOP-Bang score of all, males and females are distributed close to the mean STOP-Bang score of each group. Predictive parameters of STOP-Bang scores were analyzed first as the entire group together and secondly by sex. Descriptive summary statistics for the participants’ population including summary score for STOP-Bang score and AHI are displayed in Table 1. The responses to tired/sleepy and history of hypertension were reported more frequently by females but loud snoring and observed apneas were no different between the sexes. Age was similar in both sexes, but females had a higher BMI; however, males had a greater neck size and a higher AHI value.

The number of participants in each category of sleep apnea severity (AHI) versus the STOP-Bang scores are shown in Table 2. In this study, no individual reported the STOP-Bang score of 8.

**Figure 2:**
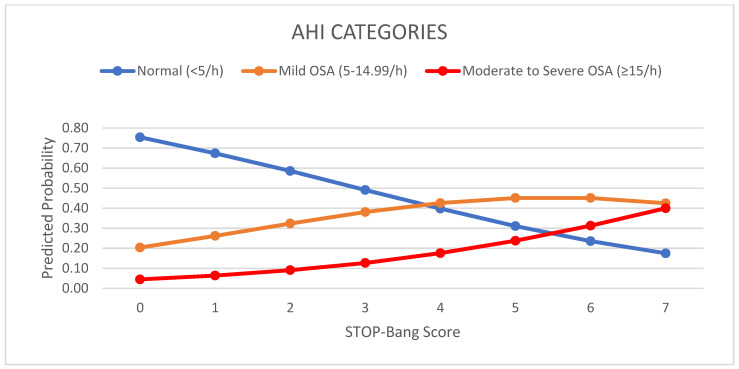
Predicted probability of sleep apnea severity based upon a linear model of the total population studied versus summary STOP-Bang scores (Numerical sum of the positive responses of the STOP-Bang model questionnaire).

The predicted probabilities of sleep apnea severity based upon the ordinal regression model of the total population studied versus the STOP-Bang score are shown in Figure 2. When the composite score was zero, there was a 75.4% probability of having no sleep apnea and small probability of having moderate or severe sleep apnea (4.5%). There was a 20.4% probability of having mild OSA with a zero score, which reflects the false-negative rates. With each incremental increase in the score from 0 to 3, the probability of having no sleep apnea diminished, while the probability of having mild or moderate/severe sleep apnea increased. With any score ≥ 3, the probability continuously increased for having moderate/severe sleep apnea, while the probability for having no sleep apnea decreased. The probability of having mild sleep apnea showed no such patterns.

The sensitivity, specificity, PPVs, NPVs, and the area under the receiver operating curves (AUC) are summarized in Table 3. The AUC was 0.61 (95% CI: 0.53–0.69) and 0.67 (95% CI: 0.56–0.78) for all OSA and moderate-to-severe OSA with a STOP-Bang score ≥ 3, respectively. The confidence intervals do not include 0.5 confirming the diagnostic ability of the STOP-Bang score ≥ 3 cut-off. The STOP-Bang score ≥ 4 and STOP-Bang score ≥ 5 do not show the diagnostic ability for all OSA as the confidence intervals included 0.5; however, those cut-offs were able to show the diagnostic ability for moderate-to-severe OSA. The single question “loud snoring” and combined question “loud snoring and obese” also have a very similar diagnostic ability but lower than the STOP-Bang score ≥ 3 cut-off for all OSA, revealing that the score with all its components achieved more accurate results.

As the STOP-Bang score cut-off increased from 3 to 5, the sensitivity decreased from 53.1% to 13.3% for all OSA and the sensitivity decreased from 72.0% to 36.0% for moderate-to-severe OSA. As the STOP-Bang score cut-off increased from 3 to 5, the specificity increased from 68.4% to 93.4% for all OSA and the specificity increased from 62.6% to 93.7% for moderate-to-severe OSA. A STOP-Bang scored 3 or greater for all OSA and moderate-to-severe OSA had moderate sensitivity and specificity. The AUC was >0.60 for different severities of OSA with the highest for moderate-to-severe OSA at 0.67. The STOP-Bang score ≥ 3 also had high discriminative power to exclude moderate-to-severe OSA as reflected by the NPV of 94.4%. The STOP-Bang score ≥ 3 had a higher sensitivity compared to the symptom of loud snoring or loud snoring plus obesity; however, it had a lower specificity compared to the symptom of loud snoring, or loud snoring plus obesity in this population.

The sensitivity, specificity, PPVs, NPVs, and the area under the receiver operating curves (AUC) by sex are summarized in Table 4. The AUC was 0.64 (95% CI: 0.52–0.76) and 0.77 (95% CI: 0.65–0.88) for all OSA and moderate-to-severe OSA with a STOP-Bang score ≥ 3, respectively, for males and the AUC was 0.58 (95% CI: 0.48–0.68) and 0.57 (95% CI: 0.39–0.75) for all OSA and moderate-to-severe OSA with a STOP-Bang score ≥ 3, respectively, for females. The confidence intervals do not include 0.5 confirming the diagnostic ability of the STOP-Bang score ≥ 3 cut-off for males, but not for females. The STOP-Bang score ≥ 4 and STOP-Bang score ≥ 5 did not show the diagnostic ability for all OSA for both males and females as the confidence intervals included 0.5; however, those cut-offs were able to show the diagnostic ability for moderate-to-severe OSA for males. The single question “loud snoring” had a very similar diagnostic ability as the STOP-Bang score ≥ 3 cut-off for all OSA and moderate-to-severe OSA for both males and females.

As the STOP-Bang score cut-off increased from 3 to 5, the sensitivity decreased from 60.0% to 15.6% for all OSA and the sensitivity decreased from 92.3% to 46.2% for moderate-to-severe OSA for males. Similarly, when the STOP-Bang score cut-off increased from 3 to 5, the sensitivity decreased from 47.2% to 11.3% for all OSA and the sensitivity decreased from 50.0% to 25.0% for moderate-to-severe OSA for females. As the STOP-Bang score cut-off increased from 3 to 5, the specificity increased from 67.4% to 93.0% for all OSA and the specificity increased from 61.3% to 94.7% for moderate-to-severe OSA for males. Similarly, when the STOP-Bang score cut-off increased from 3 to 5, the specificity increased from 68.9% to 93.2% for all OSA and the specificity increased from 63.5% to 93.0% for moderate-to-severe OSA for females. When the STOP-Bang scored 3 or greater for both, all OSA and moderate-to-severe OSA had moderate specificity for both males and females. For males, sensitivity was moderate for all OSA and high (92.3%) for moderate-to-severe OSA. However, for females, sensitivity was low (≤50%) for all OSA and moderate-to-severe OSA. The AUC was >0.60 for different severities of OSA with the highest for moderate-to-severe OSA at 0.77 for males. The STOP-Bang score ≥ 3 also had high discriminative power to exclude moderate-to-severe OSA as reflected by the NPV of 97.9% for males. The STOP-Bang score ≥ 3 had a higher sensitivity for males compared to the symptom of loud snoring or loud snoring plus obesity; however, it had a lower specificity compared to the symptom of loud snoring or loud snoring plus obesity in this population. For females, all the STOP-Bang score cut-offs and the symptom of loud snoring or loud snoring plus obesity had no discriminating ability to diagnose participants with and without the disease/condition.

## 3. Discussion

To date, this is the first study, to our knowledge, of the validity of the STOP-Bang questionnaire in First Nations peoples in Saskatchewan, Canada. These data suggest that a STOP-Bang score ≥ 5 will allow healthcare professionals to identify individuals with increased probability of moderate-to-severe OSA, with a high specificity (93.7%) and NPV (91.8%). Analysis by sex suggested that a STOP-Bang score ≥ 5 will allow healthcare professionals to identify individuals with increased probability of moderate-to-severe OSA, with a high specificity (>90.0%) and NPV (90.0%%) for males; however, there is no diagnostic test accuracy for females.

We found that the STOP-Bang questionnaire with a cut-off score ≥ 3 has a moderate AUC of 0.61 in detecting OSA in First Nations peoples. Since the AUC is >0.5, this has sufficient diagnostic accuracy [10,11]. Our findings for the AUC were different (smaller in numbers and ranged from 0.53 to 0.61) to those reported by other studies that validated the STOP-Bang questionnaire with a cut-off score ≥ 3 in the general population [9], clinical populations [8,12], and different geographic regions [13]. Chen et al. [9] reported that the AUC for the general population was 0.73 for all OSA. Chung et al. [12] reported that the AUC for the preoperative clinic population was 0.65 for all OSA and Hwang et al. [8] reported that the AUC for patients with cardiovascular risk factors was 0.86. The STOP-Bang questionnaire with a cut-off score ≥ 3 had an AUC ranging between 0.72 in North America, 0.78 in Europe, and 0.76 in South or Southeast Asia for detecting all OSA [13]. Additionally, one study used Level I polysomnography [12] and other studies used both polysomnography and Level 3 home sleep apnea testing [8,9,12]. Our study reported moderate sensitivity and specificity values for all OSA and moderate-to-severe OSA for a STOP-Bang cut-off score ≥ 3. The STOP-Bang score was modestly superior to the symptom of loud snoring or loud snoring plus obesity in this population. Compared with other studies [8,9,12,13] where sensitivity ranges from 73% to 95% and specificity ranges from 24% to 66% for all OSA, the current study has low sensitivity (53.1%) and high specificity (68.4%). Compared with other studies [8,9,12,13] where sensitivity ranges from 68% to 97% and specificity ranges from 11% to 45% for moderate-to-severe OSA, the current study has low sensitivity (72.0%) and high specificity (62.6%).

Our results are from a First Nation general population, while most of the other studies are from clinical populations. This is important because detecting and treating OSA early is important to treat comorbidities from OSA. The results revealed that the STOP-Bang screening tool method is useful in OSA detection in this First Nation general population. A valid, simple, easily accessible, and easy-to-administer screening tool could be utilized by family doctors and assist in the early detection and treatment of OSA, prior to sleep studies in First Nations peoples.

### Strengths and Limitations

To our knowledge, this was the first study to evaluate the STOP-Bang questionnaire among First Nations peoples in Saskatchewan. The participation in a Level 3 one-night home sleep test was 56% (233/418) of those who completed the survey questionnaire. Overall, this was a population with mild OSA (34.0%) with 25 (11.6%) of them having an AHI ≥ 15 (moderate-to-severe OSA). Determination of a minimum sample size required for the evaluation of both sensitivity and specificity of a screening or diagnostic test is dependent upon various pre-specified parameters. The rule of thumb is to obtain a large sample, which is reasonable since it will always increase the accuracy of the estimation process. According to Bujang and Adnan [14], a sample size of 300 participants was suggested as sufficient to evaluate both the sensitivity and specificity of most screening or diagnostic tests. However, the sample size of this study was moderate (*n* = 215) which may have produced imprecise predictive parameters. Additionally, small sample sizes for subgroup analysis by sex (male = 88 and females = 127) may have produced imprecise predictive parameters and should be interpreted cautiously. Standard polysomnography (PSG) in a laboratory to detect OSA was not conducted. This study was confined to the Level 3 Home Sleep Test, which tends to underestimate the severity of OSA related to its operating characteristics and exclusive dependence on oxygen desaturation for scoring. About half of the study population was obese (49.4%). Therefore, these data would not be applicable to the general population or even another First Nation community. A previous study demonstrated that four or more hours of evaluation time provided the most accurate testing [15]. One of the limitations in this study was some of the AHI evaluation recording times were less than four hours as these tests were self-monitored. However, the mean AHI values were not significantly different from those that were four hours and greater (*p* > 0.05).

## 4. Materials and Methods

### 4.1. Study Sample

The baseline survey of the First Nations Sleep Health Project (FNSHP) was completed between 2018 and 2019 in collaboration with two Cree First Nation communities (Community A and Community B) in Saskatchewan, Canada. The methods were presented elsewhere [16,17,18] and are briefly described here. The overall goal of the FNSHP was to study the relationships between sleep disorders, risk factors and co-morbidities, and to evaluate local diagnosis and treatment. A Certificate of Approval was obtained from the University of Saskatchewan’s Biomedical Research Ethics Board (Certificate No. Bio #18-110). In addition, adherence to Chapter 9 (Research Involving the First Nations, Inuit, and Mètis peoples of Canada) in the Tri-Council Policy Statement: Ethical Conduct for Research Involving Humans was undertaken [19]. Individual participants provided written informed consent to participate in this research collaboration.

### 4.2. Data Collection

Research assistants consisting primarily of university-level students whose families were residents in the communities were hired and trained to conduct the baseline questionnaire surveys in their respective community. Data were collected via interviewer-administered questionnaires (in Community A and B) and clinical assessments (only conducted in Community A). The survey collected information on demographic variables as well as individual and contextual determinants of sleep health. The license to use the STOP-Bang questionnaire was obtained (License No. UHN# 2022-0365) from the Commercialization Unit at University Health Network, Toronto, Ontario, Canada. The STOP-Bang questionnaire was administered prior to the Level 3 home sleep test by a research assistant along with a survey questionnaire. Objective clinical measurements included a Level 3 home overnight sleep test and actigraphy. This manuscript is based on data from the questionnaires and one-night home sleep tests collected in Community A.

#### 4.2.1. Measurements

Anthropometric measurements (abdominal girth, neck circumference, hip circumference, height and weight) were obtained. Height was measured against a wall using a fixed tape measure with participants standing in stockings on a hard floor. Weight was measured using a calibrated spring scale with participants dressed in indoor clothing. Using clinical measures of weight and height, body mass index (BMI) was calculated based on the equation of BMI = weight (kg)/(height (m))^2^ [20]. In addition, a blood oxygen level using a Pulse Oximeter (Contec Medical Systems, Qinhuangdao, China) and two blood pressure measurements using the LifeSource^(^^®^ digital blood pressure monitor (A&D Medical, San Jose, CA, USA) were recorded.

Level 3 home sleep assessments were obtained by sending Level 3 overnight sleep study kits home with instructions for participants who presented for clinical measurements. Testing was conducted using the ApneaLink Air^TM^ ResMed system (ResMed, SanDiego, CA, USA). Continuous variables derived from the Level 3 studies were: apnea/hypopnea index (AHI); oxygen desaturation index; lowest and average oxygen saturation; obstructive index; central index; hypopneas; and apneas. Trained research assistants prepared the ApneaLink Air device connecting all accessories (nasal cannula, effect sensor, oximeter and belt) before giving it to the participants. The research assistants instructed the participant on appropriate use during an initial fitting to personalize the equipment settings. A participant’s instruction sheet was given to each participant to take home to assist in wearing the equipment. Once the kit was returned, the research assistants downloaded the results and checked to see if the test had been properly recorded. Upon successful completion of the test, participants were provided with a CAD 50 honorarium for completing the survey questionnaire and one-night home sleep test.

#### 4.2.2. Definitions

In adherence to the American Academy of Sleep Medicine (AASM)’s Position Statement, obstructive apnea was defined as a reduction in airflow of 90% in the presence of thoracoabdominal movements for a period of at least 10 s [15]. Obstructive hypopnea was defined as a decrease of 30% or more in the sum of thoraco-abdominal movements for at least 10 s associated with a decrease in oxygen saturation of at least 3% [21,22]. The Apnea Hypopnea Index (AHI), the number of apneic and hypopneic events per hour of sleep, was used to indicate the severity of OSA. Based on the AHI, the severity of OSA was classified as follows: none/normal—AHI <5 per hour; mild OSA—AHI ≥ 5, but <15 per hour; moderate OSA—AHI ≥ 15, but <30 per hour; or severe OSA—AHI ≥ 30 per hour [23]. For the analysis, moderate and severe OSA groups were combined due to the small number of cases in the severe OSA group. Normal, overweight, and obese were defined as BMI ≤25 Kg/m^2^; BMI = 25–29.9 Kg/m^2^; and BMI ≥ 30 Kg/m^2^, respectively.

Most clinical tests are used to classify patients as “positive” or “negative” depending on the presence or absence (respectively) of a particular sign or symptom, which is then presumed to be indicative of the presence or absence of the condition (i.e., a “positive” test result indicates that the patient has the condition) [24]. It is necessary to use evaluation measures or metrices to assess the performance of the diagnostic tests [12]. The definitions of evaluation measures (sensitivity, specificity, positive predictive value, negative predictive value, and area under the curve) are given below.

The *sensitivity* of a clinical test is the proportion of participants with the condition who are correctly identified by the test and provide a “positive” result [25,26,27,28]. If the sensitivity is high, a “negative” test result will effectively rule out the condition [26].

The *specificity* is the proportion of participants without the condition who are correctly identified by the test and provide a “negative” result [25,26,27,28]. If the specificity is high, a “positive” test result will effectively rule in the condition [26].

The *positive predictive value* is the proportion of participants with a “positive” test result who are correctly diagnosed.

The *negative predictive value* is the proportion of participants with a “negative” test result who are correctly diagnosed [25,26,27,28]. The calculations for these parameters are based on constructing a 2 × 2 contingency table, as illustrated in following table [24].


Condition-OSA

PresentAbsentTest ResultsPositiveTPFPNegativeFNTN

TP = “True Positives”;
FP = “False Positives”;
FN = “False Negatives”;
TN = “True Negatives”;
Sensitivity = TP/(TP + FN);
Specificity = TN/(FP + TN);
Positive predictive value (PPV) = TP/(TP + FP);
Negative predictive value (NPV) = TN/(FN + TN).

A receiver operating characteristic (ROC) curve is a plot of the sensitivity versus (1 – specificity) of a diagnostic test. The different points on the curve correspond to the different cut-points used to determine whether the test results are positive. An ROC curve can be considered as the average value of the sensitivity for a test over all possible values of specificity or vice versa [29]. Area under the ROC curve (AUC) is an effective way to summarize the overall diagnostic accuracy of the test. It takes values from 0 to 1, where a value of 0 indicates a perfectly inaccurate test and a value of 1 reflects a perfectly accurate test [29]. In general, an AUC of 0.5 suggests no discrimination (i.e., ability to diagnose patients with and without the disease or condition based on the test), 0.7 to 0.8 is considered acceptable, 0.8 to 0.9 is considered excellent, and more than 0.9 is considered outstanding [10,11]. A value of 0.5 for AUC indicates that the ROC curve will fall on the diagonal (i.e., 45-degree line) and, hence, suggests that the diagnostic test has no discriminatory ability. ROC curves above this diagonal line are considered to have reasonable discriminating ability to diagnose participants with and without the disease/condition [11].

### 4.3. Statistical Analysis

Statistical analyses were performed using IBM SPSS software (IBM Corp, Released 2020. IBM SPSS Statistics for Windows, Version 27.0. Armonk, NY, USA: IBM Corp.). The participant characteristics are presented with descriptive statistics; mean, standard deviation and range for continuous data; and frequency and percentage were used for categorical data. The primary outcome measure was the severity of sleep apnea based upon the AHI as categorized into the 3 groups of normal, mild, and moderate-to-severe. A proportional odds ordinal logistic regression analysis [30,31] was conducted using the total score of the STOP-Bang as the independent variable with AHI as an ordinal outcome. Based on the ordinal logistic regression model, predicted probabilities for each STOP-Bang score for different AHI cut-offs were calculated and plotted.

The parameters of sensitivity and specificity are important parameters when selecting a screening tool [8]. To assess the performance of the STOP-Bang questionnaire [12]., multiple 2 × 2 contingency tables were used to calculate sensitivity, specificity, positive predictive values (PPVs), negative predictive values (NPVs), and area under the curve (AUC) to assess the validity of the STOP-Bang questionnaire for different AHI cut-offs: AHI ≥ 5 and AHI ≥ 15 events per hour [23]. Choosing an appropriate cut-off value is of utmost importance in using a diagnostic test effectively [32]. Different cut-offs ≥ 3, ≥4, ≥5, a single question of loud snoring and two combined questions of loud snoring and obese of the STOP-Bang questionnaire were considered. The AUC helps to estimate how high the discriminative power of a test is. The AUC ranges from 0 and 1 and a perfect diagnostic test has an AUC of 1.0, whereas a non-discriminating test has an area of 0.5. If the AUC < 0.5, the diagnostic accuracy is not useful [10,11].

## 5. Conclusions

In summary, the STOP-Bang questionnaire is a suggestive screening tool for detecting OSA in First Nations peoples. The moderate sensitivity, specificity, PPV and NPV of the STOP-Bang questionnaire help to assess risk stratification and to facilitate the diagnosis and treatment of OSA. It will be important to carry out a future study to validate this screening tool for detecting OSA in First Nations peoples.

## Figures and Tables

**Table 1:** Summary Statistics of the participant population.

Parameter	All	Males	Females	*p* Value ^4^
N	215	88	127	
Loud snoring ^1^ (%)	28.8	27.3	29.9	0.673
Tired/Sleepy ^1^ (%)	67.4	54.5	76.4	0.001
Observed apnea ^1^ (%)	20.5	19.3	21.3	0.729
Blood pressure ^1^ (%)	19.5	12.5	24.4	0.030
BMI ^2^ (kg/m^2^)	30.9 ± 8.1 (17.3–64.1)	28.9 ± 7.3 (17.4–63.3)	32.2 ± 8.4 (17.3–64.1)	0.004
Age ^2^ (years)	37.7 ± 14.9 (18–76)	35.5 ± 14.4 (18–76)	39.3 ± 15.1 (18–73)	0.068
Neck size ^2^ (cm)	37.1 ± 5.3 (21.0–61.0)	38.2 ± 5.5 (30.0–61.0)	36.4 ± 5.1 (21.0–52.0)	0.016
Gender (%)		40.9	59.1	
STOP-Bang Score ^2^	2.5 ± 1.5 (0–7)	2.8 ± 1.4 (1–7)	2.3 ± 1.5 (0–7)	0.010
AHI ^3^	8.1 ± 11.6 (0–87.6)	9.4 ± 11.8 (0–61.4)	7.2 ± 11.5 (0–87.6)	0.168

^1^ Percent positive responses to the first 4 questions (loud snoring, tired/sleepy, observed apnea, and blood pressure) of STOP-Bang model. ^2^ Mean ± SD (Range) for BMI, age, neck size, and composite score for the STOP-Bang model. ^3^ Mean ± SD (Range) for AHI (apnea/hypopnea index). AHI is the number of apneas plus hypopneas divided by hours of total monitoring time while breathing room air. ^4^ *p* value < 0.05 considered to be significant.

**Table 2:** Number of participants with sleep apnea vs. positive STOP-Bang Model responses.

STOP-Bang Score ^1^	Apnea/Hypopnea Index		
	Normal (<5/h)	Mild OSA (5–14.99/h)	Moderate-to-Severe OSA (≥15/h)
0	5	4	1
1	33	8	4
2	42	27	2
3	18	18	6
4	11	12	3
5	5	2	5
6	3	2	2
7	0	0	2
8	0	0	0
Total	117 (54.4%)	73 (34.0%)	25 (11.6%)

^1^ Numerical sum of the positive responses of the STOP-Bang model questionnaire.

**Table 3:** Predictive parameters of STOP-Bang score, loud snoring, loud snoring and obese to screen for OSA in First Nations People.

Predictive Parameters	All OSA AHI ≥ 5/h	Moderate-to Severe OSA AHI ≥ 15/h
N	98	25
Prevalence	45.6%	11.6%
**STOP-Bang score ≥ 3**		
Sensitivity	53.1%	72.0%
Specificity	68.4%	62.6%
PPV	58.4%	20.2%
NPV	63.5%	94.4%
AUC (SE)	0.61 (SE = 0.039)	0.67 (SE = 0.056)
95% CI	0.53–0.69	0.56–0.78
**STOP-Bang score ≥ 4**		
Sensitivity	28.6%	48.0%
Specificity	83.8%	81.6%
PPV	59.6%	25.5%
NPV	58.3%	92.3%
AUC (SE)	0.56 (SE = 0.04)	0.65 (SE = 0.064)
95% CI	0.48–0.64	0.52–0.77
**STOP-Bang score ≥ 5**		
Sensitivity	13.3%	36.0%
Specificity	93.2%	93.7%
PPV	61.9%	42.9%
NPV	56.2%	91.8%
AUC (SE)	0.53 (SE = 0.04)	0.65 (SE = 0.067)
95% CI	0.45–0.61	0.52–0.78
**Loud Snoring**		
Sensitivity	37.8%	60.0%
Specificity	78.6%	75.3%
PPV	59.7%	24.2%
NPV	60.1%	93.5%
AUC (SE)	0.58 (SE = 0.039)	0.68 (SE = 0.060)
95% CI	0.50–0.66	0.56–0.80
**Loud snoring and Obese**		
Sensitivity	26.7%	39.3%
Specificity	88.3%	84.4%
PPV	65.1%	25.6%
NPV	59.5%	91.1%
AUC (SE)	0.58 (SE = 0.038)	0.64 (SE = 0.064)
95% CI	0.51–0.65	0.51–0.77

**Table 4:** Predictive parameters of STOP-Bang score, loud snoring, loud snoring and obese to screen for OSA in First Nations People by sex.

Predictive Parameters	All OSA AHI ≥ 5/h		Moderate-to-Severe OSA AHI ≥ 15/h	
	Male	Female	Male	Female
N	45	53	13	12
Prevalence	51.1%	41.7%	14.8%	9.4%
**STOP-Bang score ≥ 3**				
Sensitivity	60.0%	47.2%	92.3%	50.0%
Specificity	67.4%	68.9%	61.3%	63.5%
PPV	65.9%	52.1%	29.3%	12.5%
NPV	61.75%	64.6%	97.9%	92.4%
AUC (SE)	0.64 (SE = 0.06)	0.58 (SE = 0.052)	0.77 (SE = 0.06)	0.57 (SE = 0.09)
95% CI	0.52–0.76	0.48–0.68	0.65–0.88	0.39–0.75
**STOP-Bang score ≥ 4**				
Sensitivity	35.6%	22.6%	69.2%	25.0%
Specificity	76.7%	87.8%	77.3%	84.3%
PPV	61.5%	57.1%	34.6%	14.3%
NPV	53.2%	61.3%	93.5%	91.5%
AUC (SE)	0.56 (SE = 0.06)	0.55 (SE = 0.05)	0.73 (SE = 0.08)	0.55 (SE = 0.09)
95% CI	0.44–0.68	0.45–0.65	0.57–0.89	0.37–0.73
**STOP-Bang score ≥ 5**				
Sensitivity	15.6%	11.3%	46.2%	25.0%
Specificity	93.0%	93.2%	94.7%	93.0%
PPV	70.0%	54.5%	60.0%	27.3%
NPV	51.3%	59.5%	91.0%	92.2%
AUC (SE)	0.54 (SE = 0.06)	0.52 (SE = 0.05)	0.70 (SE = 0.09)	0.59 (SE = 0.09)
95% CI	0.42–0.66	0.42–0.62	0.52–0.88	0.41–0.77
**Loud Snoring**				
Sensitivity	40.0%	35.8%	76.9%	41.7%
Specificity	86.0%	74.3%	81.3%	71.3%
PPV	75.0%	50.0%	41.7%	13.2%
NPV	57.8%	61.8%	95.3%	91.1%
AUC (SE)	0.63 (SE = 0.06)	0.55 (SE = 0.05)	0.79 (SE = 0.07)	0.57 (SE = 0.09)
95% CI	0.51–0.75	0.45–0.65	0.65–0.93	0.39–0.75
**Loud snoring and Obese**				
Sensitivity	24.4%	30.2%	46.2%	33.3%
Specificity	95.3%	79.7%	90.7%	76.5%
PPV	84.6%	51.6%	46.2%	12.9%
NPV	54.7%	61.5%	90.7%	91.7%
AUC (SE)	0.60 (SE = 0.06)	0.55 (SE = 0.05)	0.68 (SE = 0.09)	0.55 (SE = 0.09)
95% CI	0.48–0.72	0.45–0.65	0.50–0.86	0.37–0.73

## Data Availability

The summarized data presented in this study are available on request from the corresponding author. The data are not publicly available due to the agreement with the two participating communities.

## Abbreviations

OSA	Obstructive Sleep Apnea
PSG	Polysomnography
AHI	Apnea–hypopnea index
BMI	Body mass index
NIHB	Non-insured health benefits
CPAP	Continuous positive airway pressure therapy
FNSHP	First Nations Sleep Health Project
CIHR	Canadian Institutes of Health Research
AASM	American Academy of Sleep Medicine
ESS	Epworth Sleepiness Scale
SPSS	Statistical package for the social sciences
SD	Standard deviation
OR	Odds ratio
CI	Confidence interval
PPV	Positive predictive value
NPV	Negative predictive value
ROC	Receiver operating characteristic curve
AUC	Area under the ROC curve
